# Single-cell profiling of dynamic cytokine secretion and the phenotype of immune cells

**DOI:** 10.1371/journal.pone.0181904

**Published:** 2017-08-24

**Authors:** Xingyue An, Victor G. Sendra, Ivan Liadi, Balakrishnan Ramesh, Gabrielle Romain, Cara Haymaker, Melisa Martinez-Paniagua, Yanbin Lu, Laszlo G. Radvanyi, Badrinath Roysam, Navin Varadarajan

**Affiliations:** 1 Department of Chemical and Biomolecular Engineering, University of Houston, Houston, Texas, United States of America; 2 Department of Melanoma Medical Oncology, The University of Texas MD Anderson Cancer Center, Houston, Texas, United States of America; 3 Department of Electrical and Computer Engineering, University of Houston, Houston, Texas, United States of America; University of Sydney, AUSTRALIA

## Abstract

Natural killer (NK) cells are a highly heterogeneous population of innate lymphocytes that constitute our first line of defense against several types of tumors and microbial infections. Understanding the heterogeneity of these lymphocytes requires the ability to integrate their underlying phenotype with dynamic functional behaviors. We have developed and validated a single-cell methodology that integrates cellular phenotyping and dynamic cytokine secretion based on nanowell arrays and bead-based molecular biosensors. We demonstrate the robust passivation of the polydimethylsiloxane (PDMS)-based nanowells arrays with polyethylene glycol (PEG) and validated our assay by comparison to enzyme-linked immunospot (ELISPOT) assays. We used numerical simulations to optimize the molecular density of antibodies on the surface of the beads as a function of the capture efficiency of cytokines within an open-well system. Analysis of hundreds of individual human peripheral blood NK cells profiled *ex vivo* revealed that CD56^dim^CD16^+^ NK cells are immediate secretors of interferon gamma (IFN-γ) upon activation by phorbol 12-myristate 13-acetate (PMA) and ionomycin (< 3 h), and that there was no evidence of cooperation between NK cells leading to either synergistic activation or faster IFN-γ secretion. Furthermore, we observed that both the amount and rate of IFN-γ secretion from individual NK cells were donor-dependent. Collectively, these results establish our methodology as an investigational tool for combining phenotyping and real-time protein secretion of individual cells in a high-throughput manner.

## Introduction

Although natural killer (NK) cells were classically defined as pre-activated effector lymphocytes empowered with innate cytolytic functionality, more recent data suggest that NK cells are also endowed with complex functionalities including cytokine secretion and activation of antigen presenting cells, and can thus act as a bridge between innate and adaptive immunity [1]. NK cells are of pivotal importance in the execution of antiviral and anti-tumor responses [2]. Human NK cells are identified as CD3^-^CD56^+^ cells and are typically classified into different subsets based on the relative expression of the cell surface markers CD56 (adhesion marker) and CD16 (FcγRIIIA, low-affinity Fc receptor) [3, 4]. The majority of NK cells in peripheral blood (> 90%) are the CD56^dim^CD16^+^ phenotype, which is primarily believed to be responsible for cytolytic functionality including antibody-dependent cell mediated cytotoxicity (ADCC) mediated by CD16. By contrast, the CD56^bright^CD16^-^ phenotype is the minor population in peripheral blood and is described as primarily responsible for secretion of cytokines like interferon gamma (IFN-γ) [3, 4].

The secretion of the pro-inflammatory cytokine IFN-γ is an important mechanism of defense mediated by lymphocytes. Unlike cytotoxicity that only influences the target cell that is directly conjugated to the lymphocyte, IFN-γ secretion has a more profound influence on all cells within the microenvironment via multiple mechanisms including elevated expression of HLA-class I molecules [5], induction of chemokines that can promote immune cell infiltration [6], mediation of angiostasis [7], and prevention of the outgrowth of antigen-loss variants [8]. From a clinical perspective, the secretion of IFN-γ by immune cells is likely an important contributor to the efficacy of immunotherapies including treatment with antibodies against PD-1 and CTLA-4 [9, 10]. Direct measurement of NK cell (or lymphocyte) functions at the single-cell level requires the simultaneous monitoring of multiple parameters including the cell’s phenotype, its migration and interaction with other cells, secretion of proteins, and its survival. These challenges have been tackled by measuring just a subset of these effector functions and relying on correlative studies to establish links among cellular functionalities. While multiphoton microscopy is useful for studying lymphocyte motility and cytotoxicity *in situ* or *in vivo* [11–13], the number of immune cells that can be simultaneously tracked is small and limited to the field-of-view, potentially leading to sampling bias. In contrast, *in vitro* dynamic imaging systems [14–17] may be better suited for studying the longitudinal interactions between lymphocytes and target cells at single-cell resolution and in a high-throughput manner. Microfabricated nanowell arrays are ideal for tracking both the motility and interaction between cells [14, 16, 17]. While elegant methods like microengraving [18, 19] and the single-cell barcode chip (SCBC) [20–22] have been reported for the analysis of cytokines secreted by single cells confined in such nanowell arrays, these systems require the capture of the secreted cytokine on a separate glass substrate via encapsulation thus precluding real-time dynamic measurements of cytokine secretion [22].

Here, we have developed and validated an integrated methodology that combines nanowell arrays [15, 17] and bead-based molecular sensors [22–24] for detecting cytokine secretion dynamically without the need for encapsulation of single T cells/NK cells. We used this methodology to link the phenotype of peripheral blood human NK cells with their dynamic cytokine secretion profiles. Our results demonstrate that contrary to long-term secretion that has been routinely profiled, human NK cells bearing the CD56^dim^CD16^+^ phenotype are immediate secretors (< 3 h) of IFN-γ upon stimulation. Surprisingly, both the rate and total amount of IFN-γ secretion from individual NK cells were donor-dependent parameters.

## Methods

### Human subjects statement

All work outlined in this report was performed according to protocols approved by the Institutional Review Boards at the University of Houston and the University of Texas M.D. Anderson Cancer Center (IRB# LAB06-0755).

### TILs, PBMCs, primary T cells, NK cells, and reagents

Tumor infiltrating lymphocytes (TILs) from melanoma patients were isolated and expanded as previously described [25]. Briefly, initial TIL expansion was performed in 24-well plates from either small 3–5 mm^2^ tumor fragments or from enzymatic digestion, followed by centrifugation with Ficoll-Paque PLUS (GE Healthcare Life Sciences, USA). TILs were then allowed to propagate for 3–5 weeks in TIL-complete media containing 6000 IU/mL human recombinant IL-2 (Nestlé Health Science, Switzerland). Once the desired number of TILs was achieved, Rapid Expansion Protocol (REP) was performed in which TIL was cultured together with PBMC feeder cells (1 TIL: 200 feeders) preloaded with anti-CD3 (OKT3, eBioscience) in a G-REX 100M flask until the desired number of cells were achieved and harvested. PBMC isolation from buffy coat was performed by density gradient centrifugation using either Ficoll-Paque PLUS or Lymphoprep™ density gradient medium (Stemcell Technologies, Canada). Immunomagnetic isolation of T cells from PBMC was then conducted using EasySep™ Human T Cell Enrichment kit (Stemcell Technologies, Canada). NK cell isolation from PBMC was accomplished using the RosetteSep™ Human NK Cell Enrichment Cocktail (Stemcell Technologies, Canada), as described previously [26]. **S1 Table** provides a complete listing of important reagents used in this study.

### Functionalization of beads

1 μl of ProMag™ 3 Series goat anti-mouse IgG-Fc beads (Bangs Laboratories, Inc., USA) (~2.3×10^5^ beads) in solution was washed with 10 μl of PBS and resuspended in 19.6 μl PBS (~0.05% solids). Mouse anti-human IFN-γ (1-D1K, Mabtech) was added to the beads at a final concentration of 10 μg/ml, followed by incubation for 30 min at room temperature (RT), and then washed and resuspended in 100 μl PBS.

40 μl of LumAvidin^®^ 115 microspheres (Luminex Corp., USA) (~10^5^ microspheres) in solution was washed with the same volume of PBS and resuspended in 80 μl of PBS. Biotinylated mouse anti-human IFN-γ (7-B6-1, Mabtech) was added to the microspheres at a final concentration of 10 μg/ml, followed by incubation for 1 h at RT, and was subsequently washed and resuspended in 40 μl PBS.

### PLL-g-PEG solution preparation

Poly(L-lysine) (20 kDa) grafted with poly(ethylene glycol) (2 kDa) (PLL-g-PEG) (SuSoS, Switzerland) was dissolved in 10 mM HEPES buffer at RT (final PLL-g-PEG concentration is 0.1 mg/ml). The PLL-g-PEG solution was filtered using 0.2 μm pore size syringe filter, kept at 4 ^o^C for use within two weeks of dissolution.

### ELISPOT assays

ELISPOT assays were performed with fresh PBMC and TIL as previously described [18, 27]. Briefly, microwell plates (Merck Millipore, USA) were coated with capture antibody anti-human IFN-γ (1-D1K, Mabtech) at 10 μg/ml overnight at 4°C. The next day, the plates were washed twice with PBS and blocked with complete culture medium RPMI + 10% FBS (R10) for 45 min at 37°C. Cells were prepared as follows in triplicates: (1) 4,000 PBMCs stimulated with 10 ng/ml phorbol 12-myristate 13-acetate (PMA) and 1 μg/ml ionomycin per well; (2) 4,000 TILs derived from a melanoma patient stimulated with 10 ng/ml PMA and 1 μg/ml ionomycin per well; (3) 200,000 PBMCs stimulated with 2 μg/ml CEF peptide (CEF peptide is a peptide pool consisting of 23 MHC I-restricted 8–11 mer epitopes from influenza virus, cytomegalovirus, and Epstein-Barr virus; it has been shown to elicit IFN-γ release from CD8^+^ T cells in human PBMCs of the majority of randomly selected healthy donors); and (4) 200,000 corresponding non-stimulated cells. Next, cells were incubated at 37°C/5% CO_2_ for 18 h, followed by successive washes and incubation with biotinylated anti-human IFN-γ (7-B6-1, Mabtech), extravidin-alkaline phosphatase (Sigma-Aldrich, USA) and BCIP/NBT (Sigma-Aldrich, USA) substrate. The plate was subsequently read with an ELISPOT reader (C.T.L. counter) while taking into account background measurement.

### Thin bottom nanowell array fabrication

Standard soft lithography techniques were applied for fabrication of PDMS nanowell arrays. The nanowell pattern was designed using AutoCAD (Autodesk, USA), as described previously [14, 15, 17]. The dimensions of the square well were 50 μm×50 μm, while the pitch between two adjacent wells was set to 100 μm.

The master template of the nanowell array was fabricated by standard photolithography, using SU-8 3050 (MicroChem Corp., USA) spin-coated on a 4-inch silicon wafer (WRS Materials, USA) to yield 60 μm thickness, according to manufacturer’s directions. Silanization was achieved by vapor deposition of (Tridecafluoro-1, 1, 2, 2-Tetrahydrooctyl)-1-Trichlorosilane (UCT Specialties, USA) in a vacuum desiccator chamber overnight.

PDMS (Sylgard 184, Dow Corning, USA) was mixed in 10:1 (base-to-curing agent, weight ratio), then degassed in a vacuum desiccator chamber for 1 h. 10 ml degassed PDMS mixture was poured onto the master and spun at 1000 rpm for 30 s with an acceleration of 500 rpm/s. The silicon master with PDMS thin layer was baked in a convection oven at 80 ^o^C for 3 h. After curing, the nanowell arrays in PDMS were peeled and cut to fit standard 50 mm Petri dishes.

The nanowell array was air plasma-oxidized and bonded to the bottom of 50 mm Petri dish (Ted Pella Inc., USA). Immediately prior to use, the nanowell array was re-oxidized with air plasma and then incubated with 1.5 ml PLL-g-PEG solution for 20 min at 37 ^o^C. The PLL-g-PEG solution was aspirated from the nanowell array, and the array was subsequently rinsed with R10 before use in cell-based assays.

### Finite element simulations

The system of partial differential equations to model the variation of analyte concentrations, *C* (in liquid media) and *C*_s_ (on bead surface), with time, was solved using the Transport of diluted species interface, Chemical reaction engineering module in COMSOL Multiphysics 4.1. The mass balance equation involving *C*_s_ was solved using its weak form. The relative distance between the bead and the cell within the nanowell was varied systematically across simulations. Changes in cell and bead positions, convective transport, surface diffusion on the bead (*D*_s_ = 10^−25^ m^2^/s), non-specific adsorption on walls and analyte degradation were neglected to simplify numerical simulations.

### TIMING assays for the study of NK cells phenotypes and IFN-γ secretion

Functionalized beads and pre-stained NK cells (anti-CD16-PE, 3G8, BD Pharmingen^™^; anti-CD56-biotin, HCD56, BioLegend; streptavidin-Brillant Violet™ 421, BioLegend) were loaded sequentially onto a nanowell array. The nanowell array was incubated in 1.5 ml R10 that contained 1 μg/ml detection antibody against IFN-γ (1-D1K, Mabtech) conjugated with Alexa Fluor^®^ 488 (AF488), 10 ng/ml PMA and 1 μg/ml ionomycin. The nanowell array was imaged using a ZEISS fluorescent microscope with 20× 0.8 NA objectives and a scientific CMOS camera (Orca Flash 4.0). The phenotype of the cells was imaged with 3 channels (brightfield, CD16, CD56) at the initial time point and all beads-related channels (brightfield, AF488, beads) were imaged at subsequent time points for the duration of 6 h with 10 min intervals.

### Automated image segmentation

Images at the initial time point were analyzed through in-house algorithms to acquire fluorescent intensities (FIs) of all channels (brightfield, CD16, CD56, beads) and the frequencies of cells and beads within each well. Nanowells containing single beads were chosen for further analysis. Analysis of time-lapse for beads was processed by a modified pipeline for FIs from IFN-γ channel at each time point [17]. Access and Excel (Microsoft, USA) were used for matching data between cell phenotyping and FI change of beads.

As time increased, the beads FI (IFN-γ channel) followed a sigmoidal trend. Thus, we plotted and fit FI versus time using GraphPad Prism 6 (GraphPad Software Inc., USA) using a four-parameter logistic curve fit model (log [agonist]–the concentration model [variable slope]) whose formula was rewritten in order to include all the available data points for fitting, allowing quantification of the EC50 that reflected the critical secretion time.

$$MFI=Bottom+\frac{(Top-Bottom)\times t^{h}}{t^{h}+{EC50}^{h}}$$

Bottom and Top are the corresponding values of the low plateau and high plateau, respectively; *t* is the time when the imaging was recorded during the time-lapse experiment (*t* = 0 min represents the first time point); EC50 is the time when the MFI reaches half way between Bottom and Top; *h* is the Hill slope.

## Results

### Thin bottom nanowell arrays

As we and others have previously reported, nanowell arrays fabricated in PDMS offer a convenient route to track the dynamic functional behavior of immune cells but might not be amenable to high-resolution imaging due to the thickness of the bottom of the PDMS array [15, 17]. In order to overcome this limitation, we fabricated nanowell arrays in PDMS by spin-coating that enabled control over the thickness of the bottom of the PDMS nanowells [16, 28].

**S1A Fig** shows SEM top view images of the nanowell array obtained by spin-coating. The depth of the well was measured across multiple regions of a 10 mm×2 mm chip and confirmed by optical microscopy to be 63±2 μm (N = 136, **S1B Fig**). Similarly, the bottom thickness of the PDMS was uniform across the chip (84±2 μm, N = 205, **S1B Fig**) and this facilitated adaptation of the nanowell array to high-resolution microscopy.

**Fig 1:**
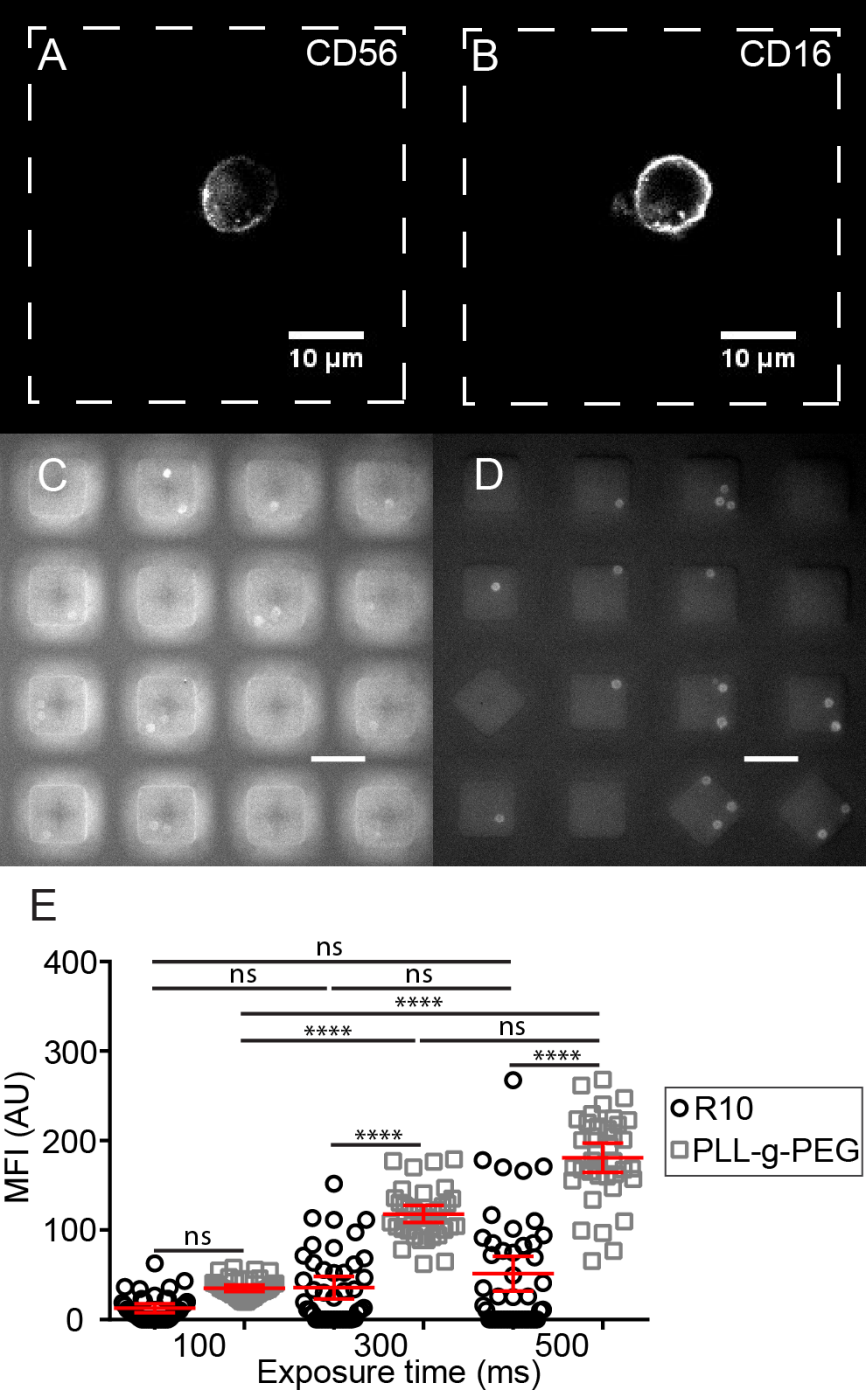
PLL-g-PEG surface modification of nanowell arrays significantly increases surface passivation. Fluorescence microscopy images of a labeled human NK cell were recorded using a 100× objective: (A) CD56 and (B) CD16. Previously frozen, human T cells isolated from peripheral blood were thawed and rested overnight, stained with anti-CD4-Brilliant Violet™ 421 on nanowell arrays either passivated with: (C) R10 or (D) PLL-g-PEG (exposure time = 500 ms). Scale bar = 50 μm. (E) Background corrected mean fluorescent intensities of individual cells in either PLL-g-PEG or R10 passivated nanowell arrays. Each dot represents a single T cell. Non-parametric tests were performed for comparison of populations corrected fluorescent intensities of CD4^+^ T cells. ****: *p*-value < 0.0001; ns: not significant; mean±SEM is shown.

To demonstrate proof-of-principle that the thin bottom nanowell arrays were compatible with high-resolution imaging, human NK cells were isolated from peripheral blood by immunodensity separation, stained with antibodies directed against the phenotypic markers CD16 and CD56, and then ~50,000 of these cells were loaded onto the nanowell array. Imaging was accomplished using Nikon confocal microscope using a 100× objective (**Fig 1A and 1B**).

### PLL-g-PEG treatment of PDMS nanowell arrays reduces non-specific binding

Despite the fact that PDMS is widely adopted for the fabrication of microfluidic devices, PDMS tends to display a high level of non-specific protein adsorption. Although a partial reduction in this effect can be accomplished by the oxidation with air plasma that renders PDMS hydrophilic, a better strategy had to be implemented since we were interested in the dynamic secretion of proteins from single cells in PDMS nanowell arrays. In order to reduce the non-specific adsorption of proteins, we explored the utility of PEG treatment of PDMS. The ability of PEG and its derivatives to passivate surfaces is well described and a graft copolymer of PEG with poly-L-lysine (PLL-g-PEG) has been previously reported for use in PDMS microchannels [29].

PDMS nanowell arrays were oxidized using air plasma to render the surface hydrophilic with silanol groups and incubated with a 100 μg/ml solution of PLL-g-PEG in HEPES. Subsequent to washing, human T cells isolated by immunomagnetic separation from PBMCs were loaded onto two separate nanowell arrays and stained with mouse anti-human CD4 antibody conjugated to Brilliant Violet™ 421 (OKT4, BioLegend). In the absence of surface modification, the signal from the cells was obscured by the background fluorescence from the nanowell edges (**Fig 1C**). By contrast, even a short 20 min treatment with PLL-g-PEG demonstrated excellent surface passivation leading to clearly distinguishable cells and very little background staining of the nanowell edges (**Fig 1D**). In order to quantify the differences arising from the signal against the background, the background corrected mean fluorescence intensities (MFI) were computed for at least 30 single cells using ImageJ (NIH, USA). Regardless of the exposure time used (100–500 ms), PLL-g-PEG-treated nanowell arrays showed consistently enhanced cell-specific labeling, and an increase in the signal with increasing exposure times (*p*-value < 0.0001, **Fig 1E**), confirming effective surface passivation. These results confirmed that even a short treatment with PLL-g-PEG was sufficient to reduce non-specific adsorption and thus all our nanowell arrays were passivated using this method.

### The frequency of IFN-γ-secreting T cells enumerated by functionalized beads within nanowell arrays is correlated to the same responses determined using ELISPOT

**Fig 2:**
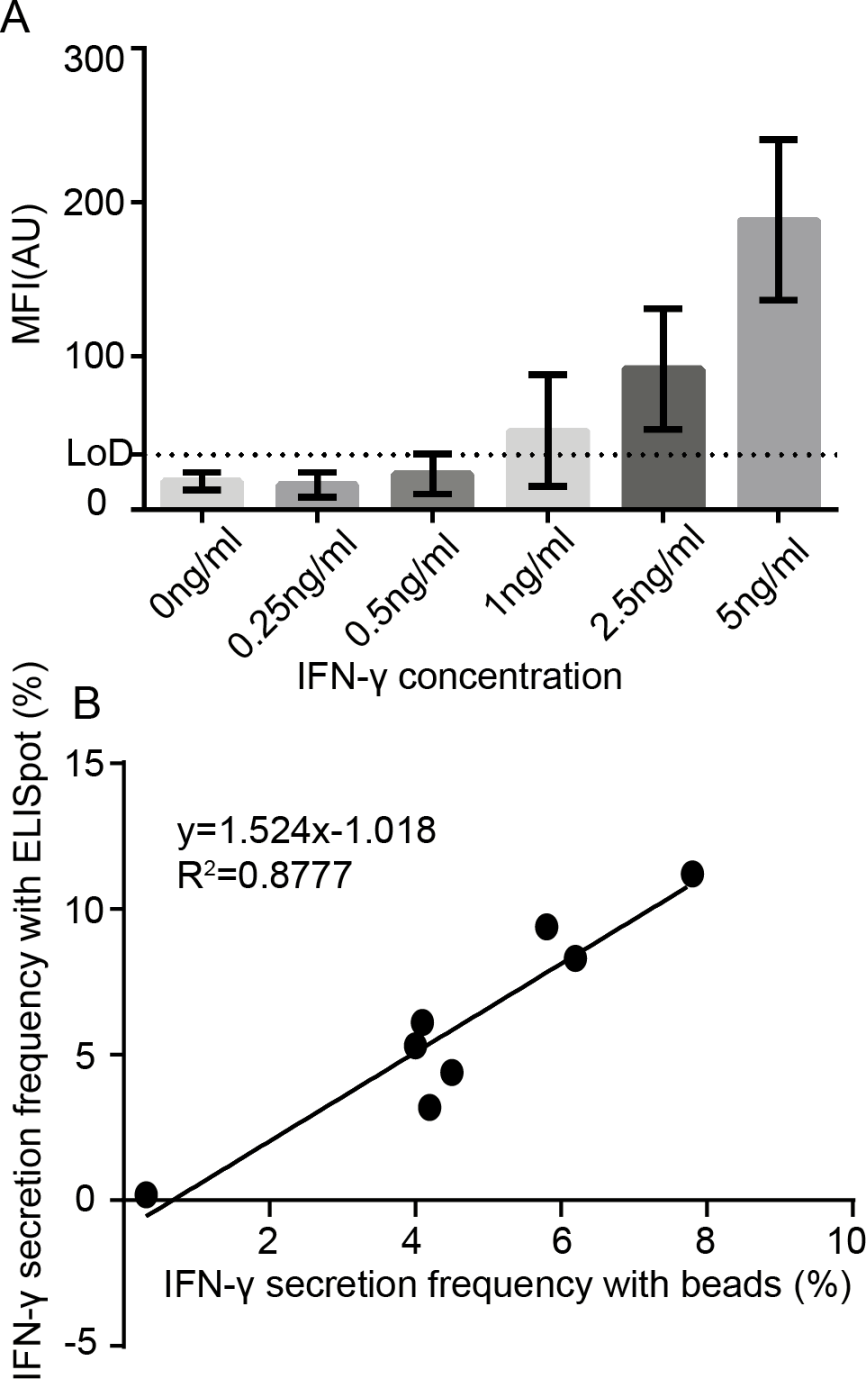
The frequency of IFN-γ-secreting T cells enumerated by functionalized beads within nanowell arrays is correlated to the same responses determined using ELISPOT. (A) Background-corrected mean fluorescence intensity (MFI) detected from a minimum of 30 IFN-γ-positive beads, as a function of IFN-γ analyte concentration with functionalized LumAvidin^®^ beads, determined on nanowell arrays. (B) Comparison of the bead assay against ELISPOT for detection of single effector cells (PBMC or TIL) secreting IFN-γ at varying level of antigenic stimulation (viral peptide pools or PMA/ionomycin). Linear regressions show that both approaches are significantly correlated (R^2^ = 0.87, *p*-value = 0.0008).

We first tested the ability of functionalized beads to efficiently capture proteins secreted by single cells after incubation in individual nanowells by measuring the limit of detection (LoD) of functionalized beads at different analyte concentrations. Antibody-coated beads were incubated with varying concentrations of IFN-γ (0.25–5 ng/ml) for a period of 2 h at 37°C, loaded onto glass bottom Petri dish, and subsequently detected with a fluorescently labeled secondary antibody. The background-corrected mean fluorescent intensity (MFI) quantified across a minimum of 30 beads confirmed that IFN-γ was detectable at a concentration of 2.5 ng/ml **(Fig 2A)**. Next, the correlation between the nanowell-encapsulated bead assay and ELISPOT for quantifying frequencies of single immune cells secreting IFN-γ upon activation was determined. To account for variations in stimulus and the diversity of T cell populations, the frequency of IFN-γ secreting single T cells was enumerated under three sets of conditions: (1) stimulation of PBMCs with PMA/ionomycin; (2) stimulation of *in vitro* expanded, melanoma TILs with PMA/ionomycin; and (3) incubation of PBMCs with HLA-class I peptide pools derived from common viral antigens (CEF peptide pool). An aliquot of 10^6^ cells was stimulated for a period of 3–5 h, from which an aliquot of ~100,000 cells was loaded onto a nanowell array. A suspension of 200,000 beads pre-coated with anti-IFN-γ (1-D1K, Mabtech) was subsequently loaded onto the nanowell array and incubated for a period of 2 h at 37°C. By analyzing an average of 10,182±8,589 (mean±SD) nanowells containing single cells matched to one or more beads, the frequency of the activated T cell IFN-γ response was determined to be 0.40–7.8%. The magnitude of these responses was similar to those recorded by ELISPOT [0.20–11.2%], and results of both assays were significantly correlated (R^2^ = 0.87, *p*-value = 0.0008), demonstrating that beads can be utilized to capture cytokine secretion from single cells (**Fig 2B)**. In the absence of stimulation, the frequency of IFN-γ beads detected when incubated with immune cells was < 1 in 10,000 and this result sets the limit of detection of our assay at 0.01%. In summary, these results established that functionalized beads within nanowell arrays were capable of detecting IFN-γ secretion from single immune cells at frequencies correlated with those from conventional ELISPOT assays.

### In open-well systems, analyte capture density increases linearly with time

**Fig 3:**
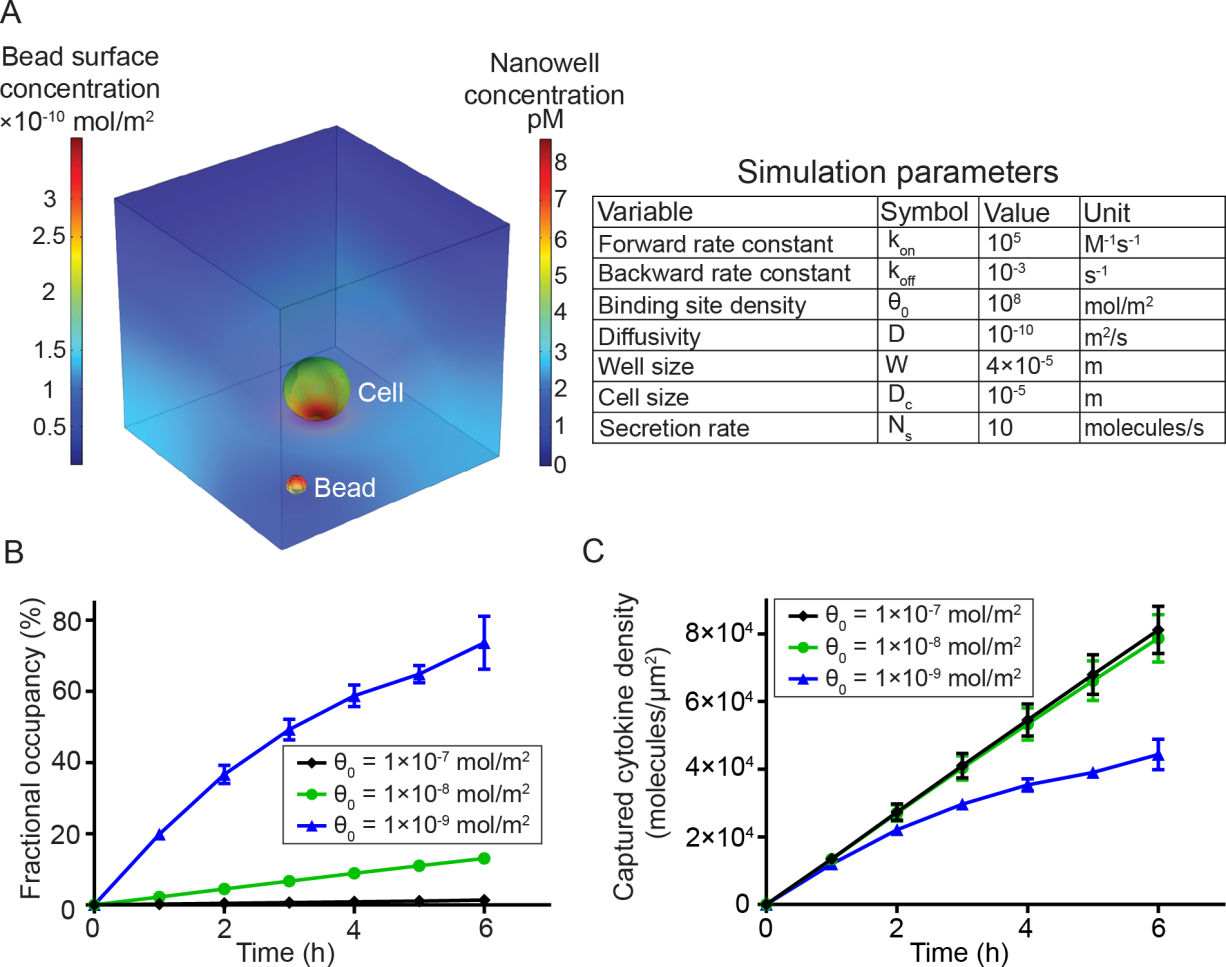
Finite element analysis to model the efficiency of capture of analyte secreted from single cells in open-well systems. (A) Snapshot of heat maps showing analyte concentration in the liquid phase across the well (right) and on the bead surface (left) after 5 h of secretion in a 40 μm nanowell. The simulation parameters are shown in the table on the right. (B) Fractional occupancy of 5 μm beads as a function of incubation time when the binding site density was varied across three orders of magnitude. For a single-cell secreting at a constant rate, beads with the lowest binding site density possess the highest fractional occupancy. Mean±SEM is shown. Error bars were determined by varying bead and cell positions relative to each other (shown only if SEM is higher than 2.5%). (C) The variation in captured cytokine density obtained by varying the density of capture antibodies on the surface of the bead; beads with higher binding site density (*θ*_0_ = 1×10^−8^ mol/m^2^, 1×10^−7^ mol/m^2^) showed more concentrated cytokine-antibody complexes on the bead surface, thus likely leading to better fluorescent pixel intensity. Mean±SEM is shown. Error bars were determined by varying bead and cell positions and weren’t shown if SEM is lower than 1800 molecules/μm^2^.

As opposed to encapsulated systems, open-well configurations can be advantageous for the long term monitoring of cell fate and function since they allow a continuous exchange of gases and nutrients. Furthermore, they avoid potential alterations of cellular behavior that can arise from the artificially high local concentrations of analytes commonly found in closed systems [30]. A disadvantage of open-well systems is that the analyte secreted by an individual cell within a nanowell is subjected to persistent diffusion into the bulk medium, potentially lowering the sensitivity. Therefore, we sought to quantify the efficiency of analyte capture on beads by modeling a simplified open-well system using finite element simulations (**Fig 3A**). The concentration of analyte in liquid media (*C*) can be described using Fick’s 2^nd^ law,

$$\frac{\partial C}{\partial t}=D\nabla^{2}C$$

where *D* represents the diffusion coefficient of the analyte. Since the walls of the PDMS can be assumed to be largely impermeable to proteins [31], the flux at these boundaries was set to zero. At a constant rate of analyte secretion from the cell (10 molecules/sec), the mass balance of analyte concentration on bead surface (*C*_s_) was determined by the equation

$$\frac{\partial C_{s}}{\partial t}=D_{s}\nabla^{2}C_{s}+k_{on}C(\theta_{0}-C_{s})-k_{off}C_{s}$$

where *D*_*s*_ represents diffusivity of the analyte on bead surface, *k*_on_ and *k*_off_ represent kinetic binding constants determined by the strength of capture antibody-analyte interaction, and *θ*_0_ represents the number of capture antibodies available per unit surface area of the bead. The choice of values for the parameters (**Fig 3A**) was based on commercially available antibody binding affinities, the known rates of cytokine secretion from lymphocytes, and previously reported numerical simulations of closed systems [31]. Initial concentrations of analyte in liquid media and bead surface were set to zero and the increase in fractional occupancy ($∯\frac{C_{s}}{\theta_{0}}$) on the bead with time as the cell secretes the analyte was modeled. Upon validating the model with previously published data [31], we sought to optimize the density of capture antibody molecules, one tunable variable to maximize captured cytokine density (and therefore the fluorescent pixel intensity). For a set bead diameter, the simulations showed that the fractional occupancy (fraction of antibodies bound by cytokines) increased when the total number of binding sites was decreased (**Fig 3B**), which is consistent with ambient analyte theory that predicts that higher sensitivity can be achieved by lowering the number of antibodies used to capture the analyte [32]. Ultimately however, the overall fluorescent signal is proportional to the density of antibody-cytokine pairs. This density is determined by both the fractional occupancy of captured cytokine and binding site density of capture antibodies. As expected, captured cytokine density increased with time (0–6 h) regardless of the density of capture antibody molecules (1×10^−9^–1×10^−7^ mol/m^2^); during short-term assays (≤ 2 h), there was not a significant difference in the various cytokine capture densities profiled. During longer assays (2–6 h), as expected, beads with smaller density of capture antibody molecules (1×10^−9^ mol/m^2^) tend to saturate cytokine capture quicker. This saturation was only observed at the lowest density of antibody molecules and subsequent increases in antibody density (1×10^−8^–1×10^−7^ mol/m^2^), did not significantly increase the density of cytokines being captured (**Fig 3C**). In summary, the results of these simulations suggested that within the short window of experimental interrogation (0–6 h), the captured cytokine density (and hence fluorescence intensity on the beads) increased linearly as a function of time. Furthermore, since the captured cytokine density was not significantly altered by increasing the antibody density on the bead, we chose to experimentally utilize beads with binding site capacities in this density range (1×10^−8^–1×10^−7^ mol/m^2^).

### An open-well system can be used to profile the dynamic secretion of cytokine molecules from individual NK cells

**Fig 4:**
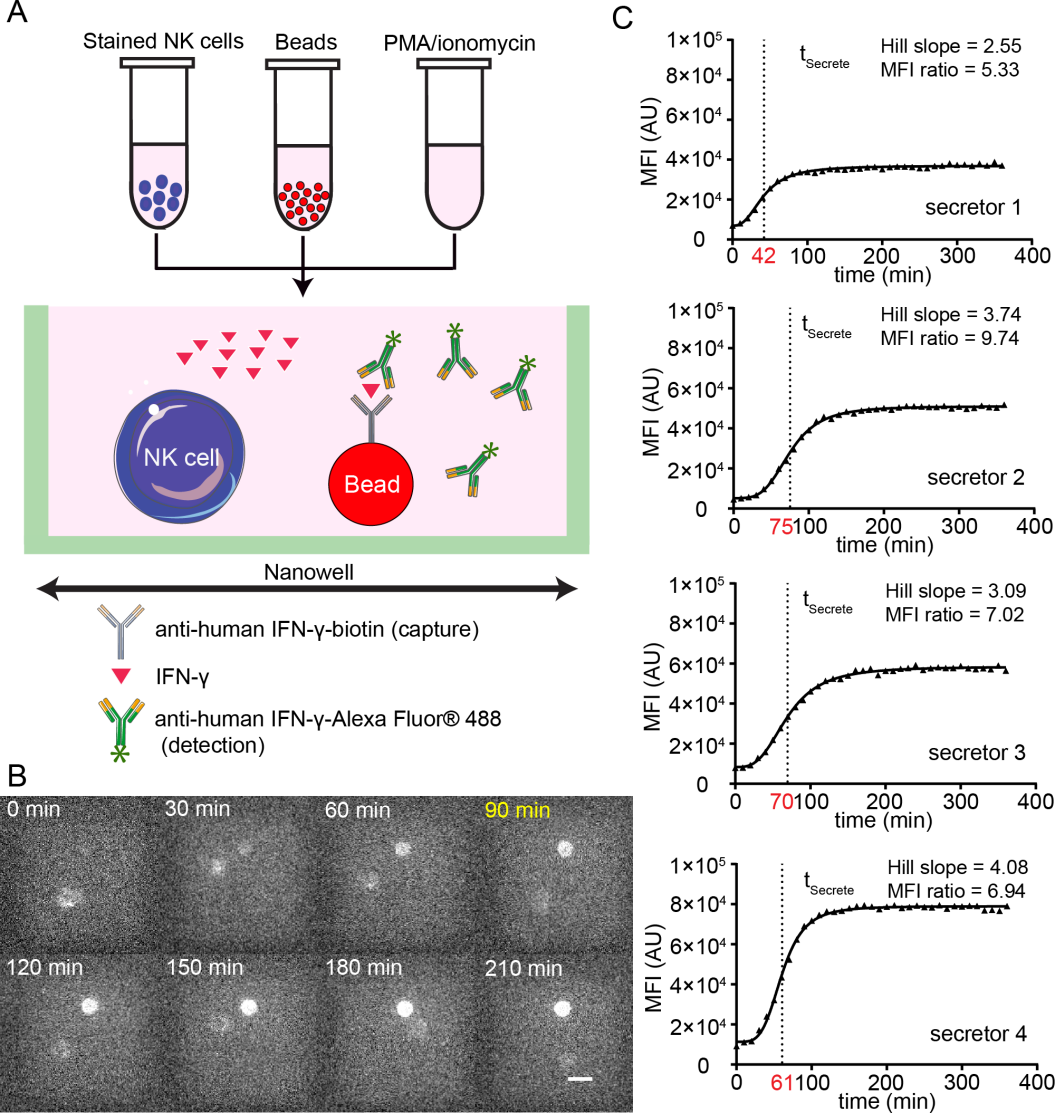
Bead-based nanowell arrays can enable monitoring the dynamic IFN-γ secretory activity. (A) Schematic of immuno-sandwich design for detecting IFN-γ secretion from single NK cells using nanowell arrays. (B) Dynamic tracking of the IFN-γ secretory activity of an NK cell within the same nanowell: t_Secrete_ is 90 min. Scale bar = 10 μm. (C) Four representative examples of dynamic fluorescence intensity (MFI) of the beads (IFN-γ secretion) upon activation of individual NK cells. The best-fitting response curve is overlaid on top of the raw data (triangles). The t_Secrete_ (red), Hill slope and MFI ratio are shown for each of the NK cells secreting IFN-γ.

Since the end-point experiments confirmed the ability to detect IFN-γ from single immune cells upon activation, and the modeling suggested that the beads should work well in an open-well system, we next wanted to investigate if dynamic secretion of IFN-γ could be detected from individual NK cells upon activation. Human NK cells isolated *ex vivo* were stained and loaded into individual wells of a nanowell array and were incubated in R10 containing the mitogenic activators PMA/ionomycin; cytokine secretion was quantified by the formation of immuno-sandwiches on beads (**Fig 4A, S2 Fig**). We modified our previously-reported image analysis algorithms to not only enable the automated segmentation and tracking of cells but to also facilitate the identification of fluorescence intensity on the beads monitoring the secretion of IFN-γ [17]. Dynamic tracking of the AF488 fluorescence demonstrated that these bead-based sensors could report IFN-γ secretion from individual NK cells incubated within the same nanowell (**Fig 4B**). Individual NK cells could be identified as secretors and non-secretors based on simple thresholding, and the fluorescence intensity of beads incubated with secretors showed a characteristic sigmoidal response that could readily be fit to a standard dose response curve to identify the characteristic time of secretion (t_Secrete_, **Fig 4C**).

### CD56^dim^ CD16^+^ NK cells are immediate secretors of IFN-γ

**Fig 5:**
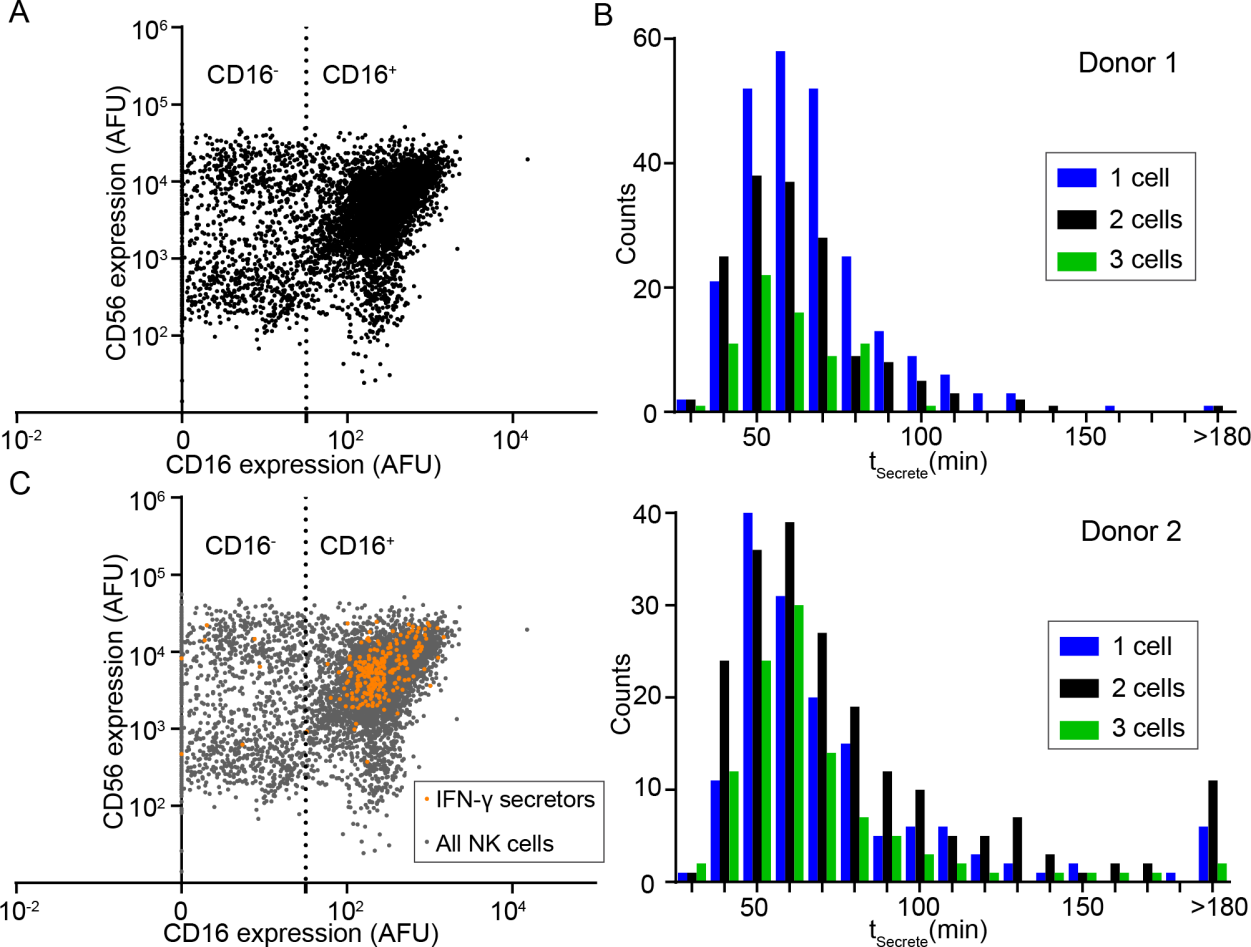
CD56^dim^CD16^+^ NK cells are immediate secretors of IFN-γ. (A) Representative phenotypic classification determined by imaging cytometry (dot plot) of NK cells based on CD16 and CD56 staining. (B) Histograms of t_Secrete_ showed a conserved pattern of distribution across two different donors. (C) In comparison to the parent population, NK cells that were immediate secretors of IFN-γ were predominantly the CD16^+^ phenotype (*p*-value < 0.0001).

Having established the feasibility of our method to detect both the phenotype and the dynamic cytokine secretion profile of individual NK cells, we next sought to define the subset of human NK cells that were immediate secretors of IFN-γ upon stimulation. Towards this objective, NK cells isolated *ex vivo* from fresh blood were enriched by immunodensity sorting, labeled with antibodies against CD16 and CD56, and loaded onto a PDMS nanowell array along with pre-functionalized beads coated with IFN-γ capture antibodies as cytokine sensors. Our phenotypic classification of NK cell subsets determined by imaging cytometry was consistent with known NK cell subsets determined by flow cytometry as previously reported (**Fig 5A**) [4]. Control nanowell arrays were set up with stained NK cells and IFN-γ sensor beads, which were imaged dynamically for a period of 6 h to confirm that the CD16 and CD56 antibodies used for immunostaining did not enable NK cells activation.

Immediately subsequent to recording the phenotype of the NK cells, the entire nanowell array was immersed in cell culture media R10 containing PMA/ionomycin to enable mitogenic stimulation. As anticipated, individual NK cells demonstrated a heterogeneous dynamic IFN-γ secretion profile, as reflected by the distributions of t_Secrete_ (**Fig 5B**). IFN-γ secretion was detected as early as 30 min from a small subset of NK cells, and the peak of the distribution of t_Secrete_ for individual IFN-γ secreting NK cells was around 50–60 min; this behavior was conserved across at least two separate donors (**Fig 5B**).

**Fig 6:**
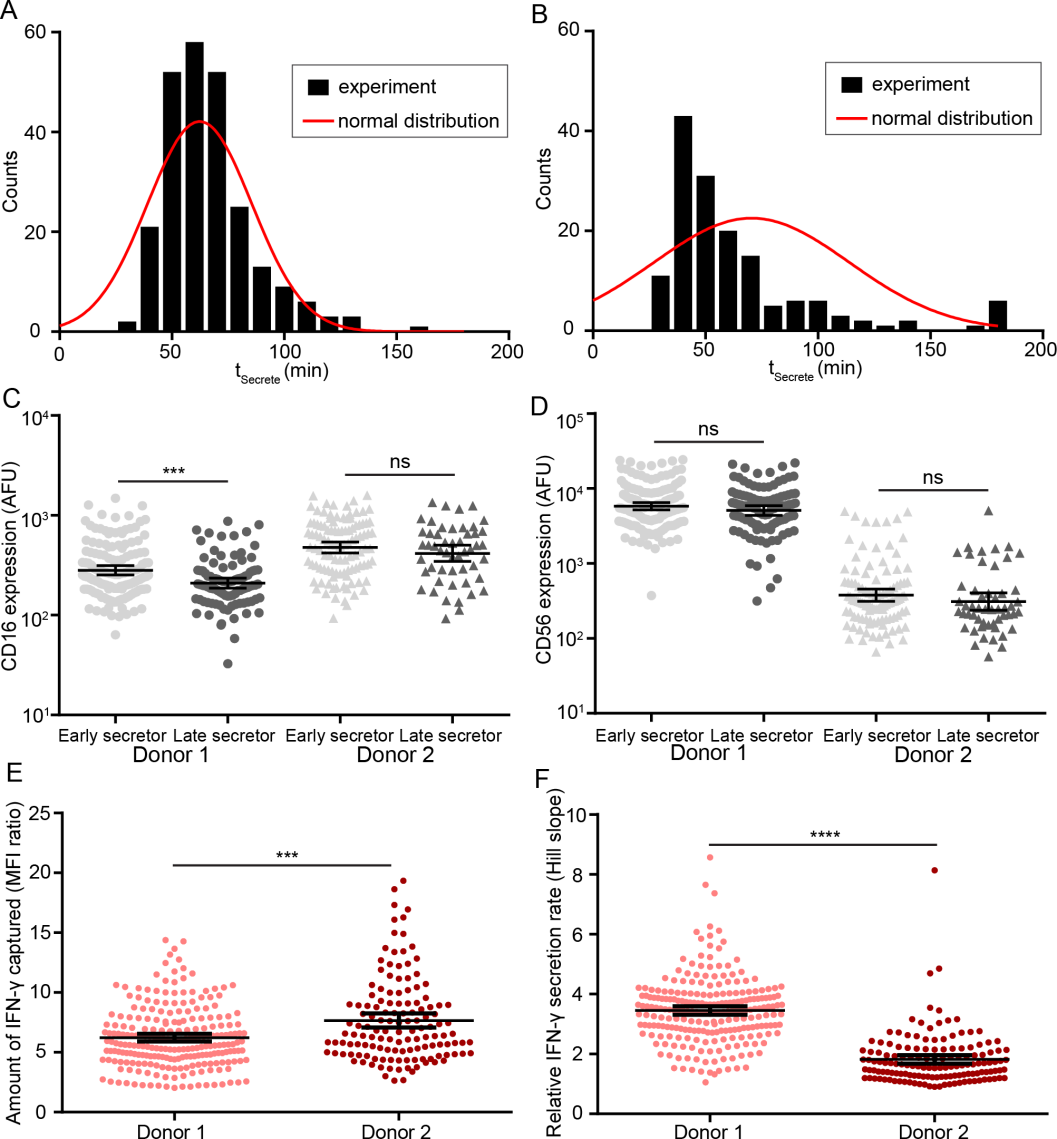
NK cells that secrete IFN-γ early have higher CD16 surface expression. (AB): The distributions (black columns) of t_Secrete_ of single-NK cells were positively skewed, indicating the existence of a faster secretor subpopulation within the population of NK cells that secrete IFN-γ. The corresponding normal distributions (red curve) were plotted using the same mean and standard deviation of t_Secrete_ of single-NK cells. The relative comparison of CD16 (C) or CD56 (D) surface expression of early secretors (t_Secrete_ < population mean) and late secretors (t_Secrete_ > population mean). (E) The amount of IFN-γ secreted by NK cells during the 6 h period of observation was statistically different across two donors. The amount of IFN-γ secreted is inferred from the ratio of fluorescent intensities (ratio of maximum and minimum value) from the fitting curve; (F) The relative IFN-γ secretion rate was a donor-dependent parameter. The rate of secretion of IFN-γ was inferred from the Hill slope (MFI versus time) obtained from curve fit on two different donors (donor 1: light red; donor 2: dark red). Error bar: mean and 95% confidence intervals are shown. Mann-Whitney test was performed, ns: not significant, ***: *p*-value < 0.001, ****: *p*-value < 0.0001.

Comparison of the phenotype of single NK cells that were immediate secretors (t_Secrete_ ≤ 180 min) to the entire parent population showed a significant enrichment of the CD16^+^ population (*p*-value < 0.0001 for donor 1 and *p*-value = 0.0034 for donor 2, **Fig 5C**). Since the distribution of t_Secrete_ (**Fig 6A and 6B**) suggested the potential existence of early secretors subpopulations within the immediate secretors, we defined early secretors and late secretors based on the mean of t_Secrete_ (donor 1: 62 min; donor 2: 70 min), and further investigated the differences in CD16 and CD56 expression of these two subpopulations. There was a trend that early secretors NK cells from both donors tended to express a higher level of CD16 on the surface (**Fig 6C**); while no similar trend was found in the comparison of expression of CD56 of early secretors and late secretors (**Fig 6D**).

To investigate other parameters besides t_Secrete_, we also compared total amount and the rate of IFN-γ secretion from individual NK cells. Consistent with the t_Secrete_, NK cell populations from a single donor tended to have individual NK cells with heterogeneous amounts and rates of secreted IFN-γ. Surprisingly, the donor with the collective population of NK cells secreting higher amounts of IFN-γ also had individual NK cells with lower rates of IFN-γ secretion (**Fig 6F**). Collectively, these results suggest that human NK cells isolated from different donors display differences in both the rate of IFN-γ secretion, likely reflective of their activation/memory state; and the total amount of IFN-γ secreted, likely reflective of the number of preformed granules containing the cytokine.

Next, the frequencies of IFN-γ secretion in nanowells that contained one, two or three NK cells were quantified to determine whether increasing NK cells density could lead to synergistic activation and faster IFN-γ secretion. Not surprisingly, increasing the number of NK cells within the nanowell increased the frequency of nanowells with IFN-γ^+^ beads (**S2 Table**). In order to investigate evidence of cooperation, we utilized the probability of single IFN-γ secreting NK cells upon activation (regardless of t_Secrete_), based on the nanowells containing exactly one NK cell. The experimentally computed frequencies for nanowells containing both 2 and 3 NK cells were lower than the theoretically computed frequencies, indicating that there was no significant evidence of cooperation or synergistic activation (**S2 Table**).

In summary, these results obtained by tracking the phenotype and dynamic secretion of IFN-γ from individual NK cells demonstrated that the NK cells classically defined as cytolytic (CD16^+^) were also immediate secretors of IFN-γ, at least upon mitogenic stimulation.

## Discussion

We have demonstrated a high-throughput assay for profiling the dynamic secretion of cytokines from individual immune cells while preserving high imaging resolution that was made possible by the fabrication of thin-bottom (<100 μm) PDMS-based nanowell arrays. This single-cell assay uses nanowell arrays for co-incubating cells with functionalized beads and thus can be readily integrated with our reported TIMING platforms to enable tracking of the key functional attributes of immune cells including phenotype, motility, cytotoxicity, and cytokine secretion; it can also serve as a front-end screen for identifying functional attributes that can be interrogated at the molecular level using multiplexed transcriptional profiling [15, 17]. Although we have demonstrated the application of this method in the context of NK cell IFN-γ secretion, the method can be adapted to other immune cells as well as other cell types for monitoring combined cellular behaviors, protein secretion, and transcriptional profiling. Furthermore, since the multiplexing of beads based on the Luminex platform [33, 34] is extensively documented, it should be straightforward to expand the number of analytes secreted by individual cells simultaneously.

PDMS is widely used in microfluidics primarily because it is low-cost, optically transparent, biocompatible and gas permeant. Despite these advantages, one of the major drawbacks of PDMS is the non-specific adsorption (NSA) of proteins onto its surface [35–37]. In dynamic imaging applications akin to what we have outlined, the NSA of both the secreted proteins and the labeled detection antibodies severely impacts both the detection limit and assay reliability/reproducibility. PEG, likely because of hydration, behaves as a hydrogel that is effective in preventing NSA [38–40]. We sought to take advantage of this property of PEG by employing a simple protocol that enables the rapid modification (20 min) of oxidized PDMS by commercially available PLL-g-PEG, in aqueous environments. We demonstrate that this simple step dramatically decreases the NSA of antibody-dye conjugates onto the surface of PDMS. Furthermore, since biotin-derivatized PEG (PLL-g-PEG-biotin) is also commercially available, this provides an avenue for surface modifying the PDMS to introduce adhesion molecules like biotinylated ICAM-1, or antibodies against the natural cytotoxicity receptors (NCRs) or CD3 to study lymphocyte activation. We have utilized our platform to profile the phenotype of human NK cells that respond quickest to stimulation. Although NK cells have been divided into two separate subsets with reciprocal functionalities—CD56^dim^CD16^+^ associated with cytotoxicity and CD56^bright^CD16^-^ with cytokine secretion—our data (tracking individual cell phenotypes with their ability to secrete IFN-γ) demonstrate that the CD16^+^ NK cells are the early secretors of IFN-γ upon activation. Our results are consistent with other correlative studies that have also suggested that the CD56^dim^ population might, in fact, be the early cytokine secretors upon activation through natural cytotoxicity receptors (NCRs) [41]. Since it has also been shown that the secretion pathway for cytokines, like tumor necrosis factor (TNF) and IFN-γ in NK cells, is distinct from the pathway used for the secretion of perforin [42], the existence of an elite population of CD16^+^ NK cells capable of both lytic and rapid cytokine secretion fits with the pivotal role of NK cells in innate immunity.

NK cells also present a clinically appealing avenue for the treatment of tumors. Since the activation of NK cells is mediated by a panel of activating and inhibitory receptors, they offer clear translational advantages. First, unlike T cells, NK cells do not require HLA typing or peptide-epitope presentation. Second, NK cells directly recognize and lyse transformed cells either due to missing HLA expression or due to the elevated expression of stress ligands [43]. Third, the translation of NK cells as drugs does not require *a priori* identification of tumor-associated antigens [44]. Additionally, the infusion of NK cells has been proven to be largely safe with no major toxicity concerns [44, 45]. The biggest disadvantage of NK cell therapies, however, has been the disappointing persistence of NK cells. With newer methods of expansion *ex vivo* [46, 47], and the ability to propagate cytokine-induced memory NK cells, these cells are poised to join the immunotherapeutic arsenal in our fight against cancers. As our work suggests, the existence of subpopulations of NK cells that are polyfunctional (CD16^+^ [cytotoxic] and IFN-γ secreting) are likely to be of keen interest in immunotherapy.

## Supporting information

S1 Fig Thin bottom nanowell arrays fabricated by spin-coating. Representative SEM images of top view (A) and side view (B) of nanowell arrays with indications of measured dimensions shown, and the summary of measurements listed (C). Scale bar = 100 **μ**m.
(TIF)

S2 Fig Distribution of functionalized beads and pre-stained NK cells in individual nanowell. Representative density matrix indicates the number of nanowells that contain 0–3 beads and 1–3 NK cells. Both numbers and frequency of wells are shown.
(TIF)

S1 Table List of reagents described in this manuscript. (XLSX)

S2 Table The frequency of IFN-γ secreting NK cells under various cell density conditions. As the cell density in the nanowell increased, frequencies of IFN-γ secreting NK cells also increased as expected, however, there was no evidence for significant cooperation or synergistic effect.
(XLSX)

S1 Movie A representative example of an IFN-γ secreting NK cell. Green denotes IFN-γ (bead) and CD16 (cell). Time is displayed as hh: mm and movie is sped up 1800×.
(MP4)
