# Supplementary figures and images for: Single-cell profiling of dynamic cytokine secretion and the phenotype of immune cells
Source: PLoS One. 2017 Aug 24;12(8):e0181904. doi: 10.1371/journal.pone.0181904 (PMC5570329; doi:10.1371/journal.pone.0181904)

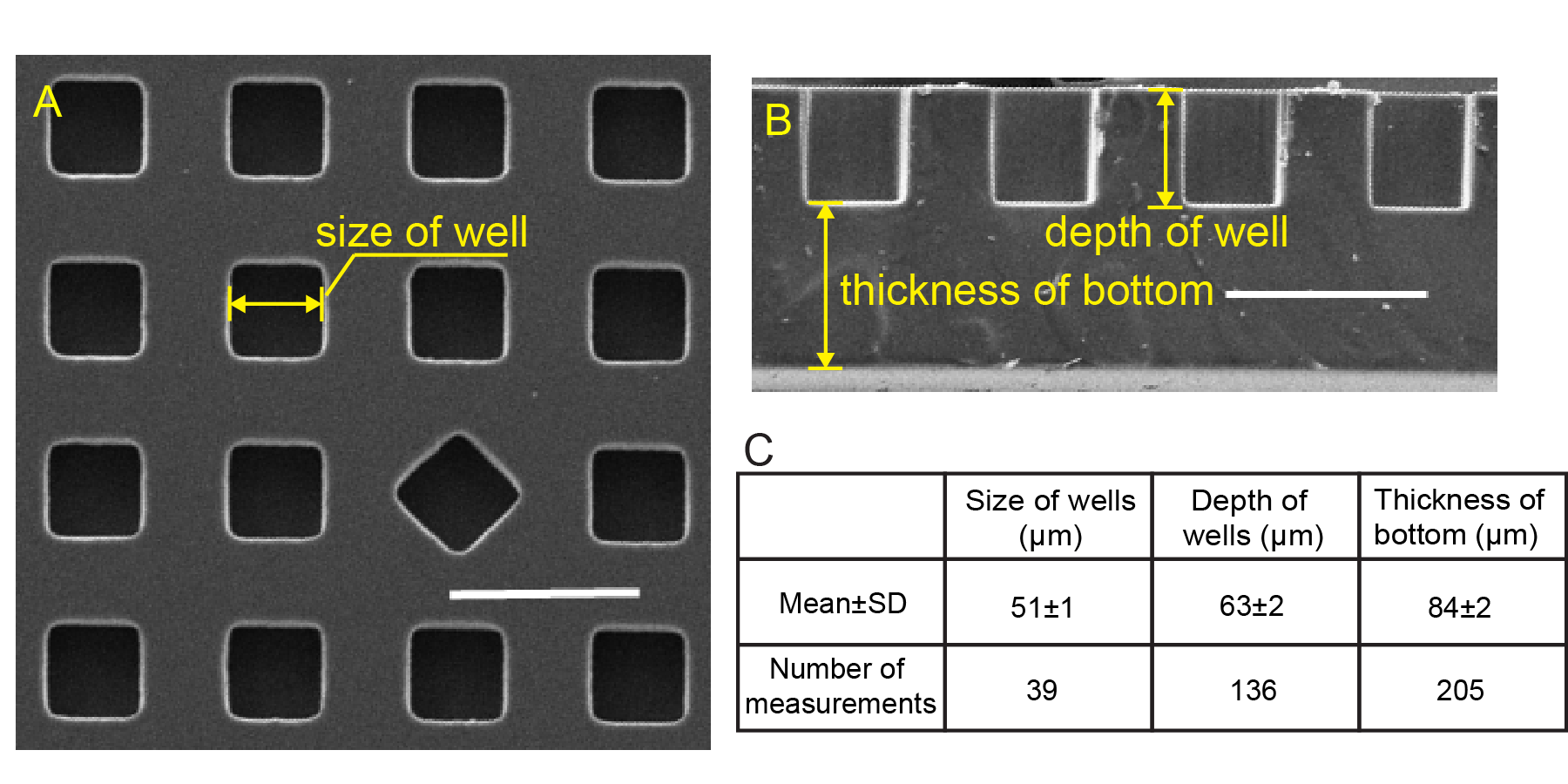

Supplement: S1 Fig — Representative SEM images of top view (A) and side view (B) of nanowell arrays with indications of measured dimensions shown, and the summary of measurements listed (C). Scale bar = 100 μm. (TIF) [file pone.0181904.s001.tif]

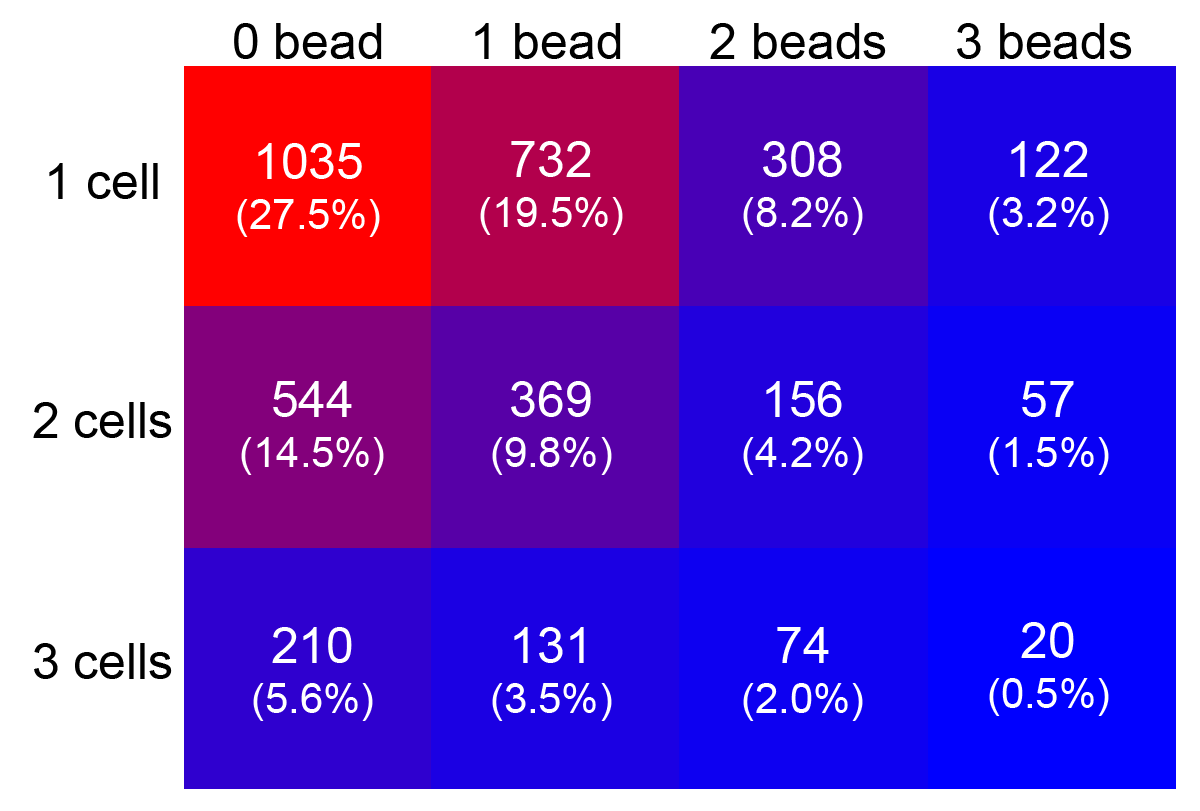

Supplement: S2 Fig — Representative density matrix indicates the number of nanowells that contain 0–3 beads and 1–3 NK cells. Both numbers and frequency of wells are shown. (TIF) [file pone.0181904.s002.tif]
